# The Potential Role of Bile Acids in Acquired Laryngotracheal Stenosis

**DOI:** 10.1002/lary.27105

**Published:** 2018-02-05

**Authors:** Adil Aldhahrani, Jason Powell, Shameem Ladak, Mahmoud Ali, Simi Ali, Bernard Verdon, Jeffrey Pearson, Chris Ward

**Affiliations:** ^1^ Institute for Cell and Molecular Biosciences Newcastle upon Tyne United Kingdom; ^2^ Institute of Cellular Medicine Newcastle University Newcastle upon Tyne United Kingdom; ^3^ Department of Otolaryngology Head and Neck Surgery, Freeman Hospital Newcastle upon Tyne United Kingdom; ^4^ Department of Otolaryngology Mansoura University Hospital, Mansoura University Mansoura Dakahlia Governorate Egypt; ^5^ Faculty of Applied Medical Sciences Taif University Turabah Saudi Arabia

**Keywords:** Bile acids, gastroesophageal reflux, laryngopharyngeal reflux, epithelial–mesenchymal transition

## Abstract

**Objective:**

Gastroesophageal reflux is thought to be a risk factor for laryngotracheal stenosis. Bile acids are a component of gastric refluxate and have previously been implicated in the development of fibrosis in other airway subsites. There is clear evidence that bile acids reflux into the upper airway. We therefore investigated the potential role of bile acids in the pathophysiology of laryngotracheal fibrosis and stenosis, specifically investigating the highly conserved process of epithelial–mesenchymal transition (EMT).

**Study Design:**

Translational research study.

**Methods:**

Human primary tracheal epithelial cells (PTECs) were challenged with the four most common digestive bile acids (cholic, chenodeoxycholic, deoxycholic, and lithocholic). EMT markers transforming growth factor (TGF)‐β1, Matrix metalloproteinase (MMP)‐9, and procollagen proteins were measured in the supernatant at 48 hours via enzyme‐linked immunosorbent assay. Real‐time polymerase chain reaction was also used to measure E‐cadherin and fibronectin expression.

**Results:**

Significantly greater concentrations of TGF‐β1 and MMP‐9 were measured in the culture supernatants of cells treated with each bile acid at 10 µmol/L. Lithocholic acid and deoxycholic acid induced significantly increased expression of procollagen protein. Upregulation of fibronectin and downregulation of E‐cadherin were observed with all bile acids, except for deoxycholic acid.

**Conclusion:**

This is the first proof of principle demonstration that physiologically relevant bile acid challenge induces EMT mechanisms in PTECs. This implies a potential role for bile acids in laryngotracheal scarring and airway remodeling of potential translational significance in laryngotracheal stenosis.

**Level of Evidence:**

NA. *Laryngoscope*, 128:2029–2033, 2018

## INTRODUCTION

Acquired laryngotracheal stenosis (LTS) most commonly occurs as a result of prolonged tracheal intubation.^1^ The subsequent airway narrowing can result in potentially life‐threatening airway obstruction.^1^ Only a small proportion of patients who undergo long‐term intubation develop LTS, and LTS can occur in patients without any history of intubation; therefore, other mechanisms of injury have been proposed.^1^, ^2^, ^3^

The relationship between gastroesophageal reflux (GER) and LTS is not fully understood; however, a number of studies have demonstrated an association.^4^, ^5^, ^6^ There is clear evidence that gastroduodenal contents are refluxed far beyond the esophagus during extraesophageal or laryngopharyngeal reflux (LPR).^7^ The three principle components of gastric refluxate are gastric acid, pepsin, and bile acids. Evidence of all three components has been found in airway bronchoalveolar lavage samples^8^, ^9^, ^10^; however, bile acids in particular are increasingly identified as critical to propagation of disease in sites distant from the stomach.^11^, ^12^

LTS is thought to involve an abnormal response of epithelial cells to injury, resulting in sustained inflammation and fibrosis. This is defined by fibroblast proliferation, collagen deposition, and ultimately the formation of scar tissue in the airway.^13^, ^14^ Activated fibroblasts are the principle mediators of tissue remodeling, and one of the key sources of fibroblast accumulation is thought to be epithelial–mesenchymal transition (EMT).^15^ In EMT, epithelial cells transform to activated fibroblasts, and transforming growth factor (TGF)‐β1 is a recognized master switch for this process.^16^ EMT is demonstrated by downregulation of epithelial markers such as E‐cadherin and increased expression of mesenchymal markers including fibronectin, matrix metalloproteinase (MMP)‐9, and procollagen from epithelial cells.^17^ We therefore investigated the mechanisms of EMT in response to an in vitro model of biliary reflux on human primary airway epithelial cells. We hypothesized that laryngotracheal epithelial cells undergo EMT in response to bile acid stimulation, representing a potential mechanism of fibrosis and ultimately LTS.

## MATERIALS AND METHODS

### Cell Culture

The appropriate ethical committee and hospital institutions granted approval for the study. Human primary tracheal epithelial cells (PTECs) from the upper airways of healthy volunteers were collected via a sheathed cytology brush. The cells were pelleted and then cultured on collagen (0.03 mg/mL)‐coated flasks in bronchial epithelial growth medium (Lonza, Allendale, NJ), supplemented with penicillin/streptomycin 100 U/mL (Sigma‐Aldrich, St. Louis, MO), incubated at 37 °C in a 5% CO_2_ incubator. Medium was changed every 2 to 3 days. Upon near confluence, cells were trypsinized (Sigma‐Aldrich), diluted in an equal volume of medium containing 10% fetal calf serum, centrifuged at 200 g for 7 minutes, and seeded in a new container

### Bile Acid Preparation and Challenge

The four major bile acids in the human digestive tract are cholic acid (CA), chenodeoxycholic acid (CDCA), deoxycholic acid (DCA), and lithocholic acid (LCA). Bile acids (Sigma‐Aldrich) were diluted in serum‐free medium to achieve the concentrations for each experiment. Bile acid solutions were incubated with cells for 48 hours at 37 °C in a 5% CO_2_ incubator. Maximal nonlethal concentration of bile acids to be used for stimulation were determined via a cell viability assay using the CellTiter‐Blue viability assay (Promega Corp. Madison, WI). Cell viability was confirmed at > 90% in each experimental condition.

### Enzyme‐Linked Immunosorbent Assays

Cell supernatant was collected and stored at −20 °C. The supernatant is the media in which the cells were growing and is a standard measurement used in cell culture studies to assay biomarkers secreted by the cells. Human TGF‐β1, Human MMP‐9, and Pro‐Collagen DuoSet enzyme‐linked immunosorbent assay (ELISA) kits (R&D Systems, Minneapolis, MN) were used according to the manufacturer's instructions.

### RNA Processing and Polymerase Chain Reaction

Human RNA was isolated from cultured cells, as directed by the RNeasy Midi Kit (Qiagen, Hilden, Germany), and the concentrations and quality were assessed by ultraviolet spectroscopy. RNA was reverse‐transcribed using the Tetro cDNA synthesis kit (Bioline, London, UK) in accordance with the manufacturer's guidelines. TaqMan primer‐probes (SensiFAST Probe Hi‐ROX Kit, Bioline) were used to determine the expression of genes of interest, E‐cadherin (Hs01023894‐m1‐CDH1) and fibronectin (Hs00365052m‐1FN1). The expression of each test gene was normalized against expression of a housekeeping gene, hypoxanthine‐guanine phosphoribosyl transferase‐1 (HPRT1) (Hs02800695‐m1).

### Statistical Analysis

Data from three technical and three biological replicates were analysed using GraphPad Prism v6 (GraphPad Software, Inc., La Jolla CA). All data are represented as mean ± standard error of the mean (SEM), and n expressed the number of repeat experiments that were performed. For experimental comparison of 3 or more groups, repeated measures one‐way analysis of variance was used, followed by Bonferroni post‐hoc test, which compares all pairs of groups. In line with convention, *P* values of ≤ 0.05 were considered significant. In this study, * refers to *P* < 0.05; ** refers to *P* < 0.01; and *** refers to *P* < 0.001.

## RESULTS

### Effect of Bile Acids on an Epithelial Marker E‐Cadherin

**Figure 1:**
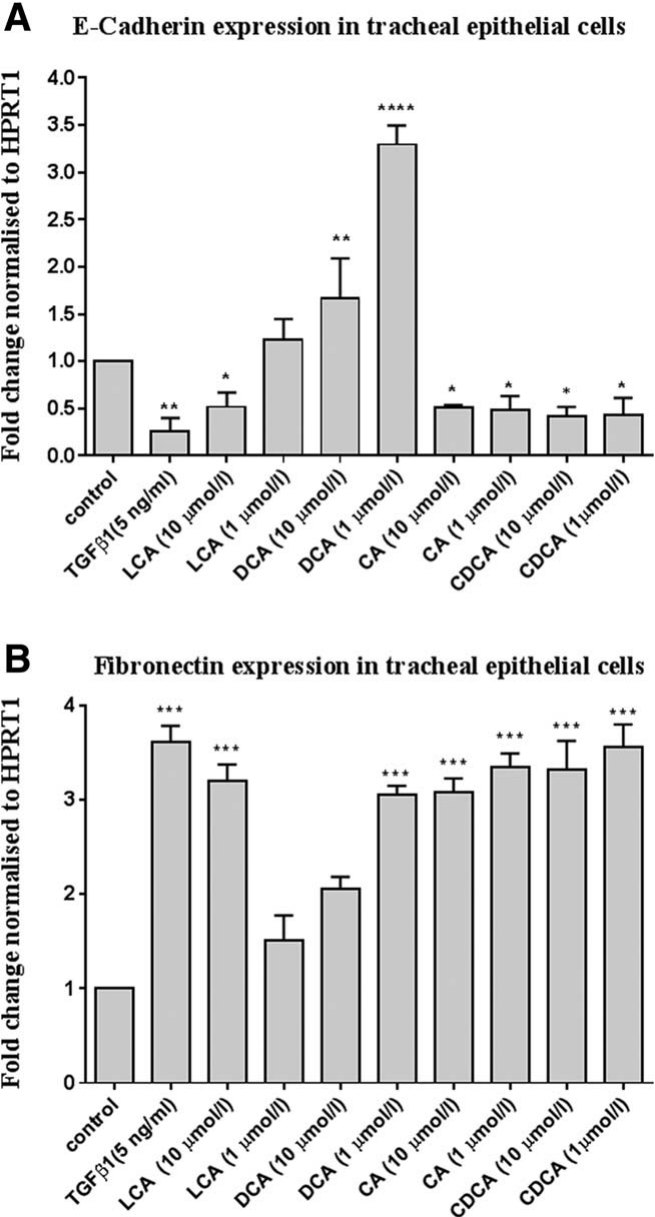
(A) E‐cadherin mRNA expression following bile acid and TGF‐β1 treatment. Confluent tracheal epithelial cells from the subglottic area were cultured for 48 hours under various conditions as follows: medium, medium containing 5 ng/mL of TGF‐β1 or 1 and 10 μmol/l of LCA, DCA, CA, and CDCA. HPRT1 mRNA was used to normalize E‐cadherin RNA mRNA levels, which were presented as mean ± SEM (n = 3). These results are from 3 similar experiments. (B) Fibronectin mRNA expression following BA and TGF‐β1 treatment. Confluent tracheal epithelial cells from the subglottic area were cultured for 48 hours under various conditions as follows: medium; medium containing 5 ng/mL of TGF‐β1 or 1 and 10 μmol/L of LCA, DCA, CA, and CDCA. HPRT1 mRNA was used to normalize fibronectin mRNA levels, which were presented as mean ± SEM (n = 3). These results are representative from 3 similar experiments. ** = *P* < 0.01, *** = *P* < 0.001, **** = *P* < 0.0001 compared to control, ANOVA. ANOVA = analysis of variance; CA = cholic acid; CDCA = chenodeoxycholic acid; DCA = deoxycholic acid; LCA = lithocholic acid; SEM = standard error of the mean; TGF = transforming growth factor.

After 48‐hour bile acid stimulation, real‐time polymerase chain reaction (RT‐PCR) analysis of E‐cadherin expression in PTECs demonstrated a significant (*P* < 0.001) decrease at 1 µmol/L and 10 µmol/L of CA and CDCA, in addition at 10 µmol/L of LCA. CA at concentration 1 and 10 µmol/L induced threefold decreases, showing no concentration‐dependency effect. CDCA at 1 and 10 µmol/L caused a twofold decrease, showing no concentration‐dependency effect. LCA at 10 µmol/L caused a twofold decrease in E‐cadherin. No significant decrease was observed in cells stimulated with 1 μmol/L LCA. Interestingly, there was significant increase at both concentrations of DCA, showing a negative concentration dependency effect (Fig. 1A).

### Effect of Bile Acids on Mesenchymal Marker Fibronectin

**Figure 2:**
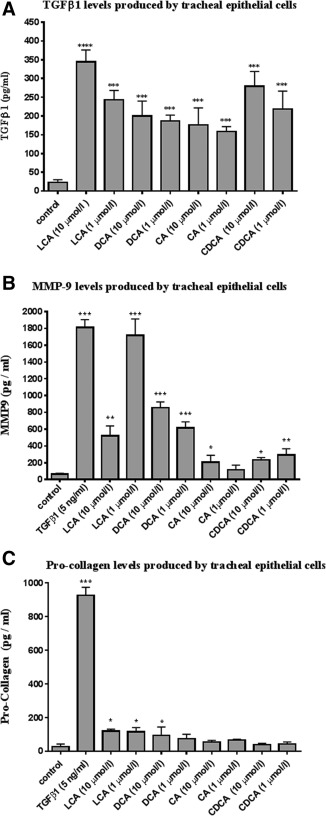
(A) ELISA of TGFβ1 levels in culture medium produced by tracheal epithelial cells from the subglottic area, cells treated with LCA, DCA, CA, and CDCA. Statistical analysis was conducted by one‐way ANOVA (n = 6). (B) ELISA of MMP‐9 production from tracheal epithelial cells from the subglottic area cell line treated with LCA, DCA, CA, and CDCA. Statistical analysis was conducted by one‐way ANOVA (n = 6). (C) ELISA of procollagen production from tracheal epithelial cells from the subglottic area treated with LCA, DCA, CA, and CDCA. Statistical analysis was conducted by one‐way ANOVA (n = 6). Bars represent mean (± SEM) values of each group. *P < 0.05, **P < 0.01, ***P < 0.001. ANOVA = analysis of variance; CA = cholic acid; CDCA = chenodeoxycholic acid; DCA = deoxycholic acid; ELISA = enzyme‐linked immunosorbent assays; LCA = lithocholic acid; MMP‐9 = matrix metallopeptidase 9; SEM = standard error of the mean; TGF = transforming growth factor.

Fibronectin expression was found to be significantly increased in PTEC, measured via RT‐PCR. It was found that CA at 1 and 10 µmol/L caused a threefold increase, and 10 μmol/L LCA caused a threefold increase in fibronectin, showing no concentration dependency effect. CDCA at 1 µmol/L caused a threefold increase, and CDCA at 10 µmol/L caused a fourfold increase in fibronectin, showing no clear concentration dependency effect. DCA at 1 µmol/L caused a threefold increase in fibronectin. No significant increase was observed in cells stimulated with DCA at 10 µmol/L and LCA at 1 µmol/L (Fig. 2B).

### Effect of Bile Acids on TGF‐β1, MMP‐9, and Procollagen Expression in Cell Supernatant

We demonstrated a significant increase in expression of TGF‐β1 in the cell supernatant of PTEC after 48 hours of exposure to all bile acids at 1 µmol/L and 10 µmol/L (Fig. 2A). Similarly, all bile acids at concentrations of 1 µmol/L and 10 µmol/L (except CA at 1 µmol/L) showed a significant increase in MMP‐9 expression in the cell supernatant (Fig. 2B). Procollagen was significantly increased after LA challenge at 1 µmol/L and 10 µmol/L and at 10 µmol/L with DCA challenge. Production of procollagen after stimulation with DCA at 1 μmol/L, CA at 1 and 10 µmol/L, and CDCA 1 and 10 µmol/L did not increase significantly. Moreover, exposure to 5 ng/mL TGF‐β1 caused a significant increase in MMP‐9 and procollagen protein production (Fig. 2C).

## DISCUSSION

We have demonstrated a potential role of bile acids in the development of EMT, a process that is implicated in airway fibrosis and subsequently in laryngotracheal stenosis (LTS). We utilized human primary tracheal epithelial cells (PTECs), challenged with levels of bile acids in the range previously shown to be physiologically relevant by Sereg‐Bahar et al. in previous work, which has shown that the mean levels of bile acids in patients with LPR were 2.1 ± 3.0 µmol/L.^10^ To our knowledge, this is the first study to investigate a possible role of bile acids in upper airway fibrosis using primary cells. The potential relationship between GER disease and LTS has been suggested by several observational studies.^4^, ^5^, ^6^ Bile acids have also been implicated in EMT in the lower airway.^11^

The identification of bile acids as a potential factor in LTS is important because this may allow novel therapeutics for the prevention or treatment of LTS to be utilized in high‐risk patients, such as people undergoing long‐term intubation. Those undergoing long‐term intubation may already be on acid suppression therapy, such as proton pump inhibitors. However, these agents may not alter the activity or concentration of bile acids in refluxate. Bile acids have a broad range of activity in nonacidic environments, making their involvement in lung pathophysiology biologically plausible.^18^ Barrier agents such as alginates or other novel targeted therapies might therefore be needed to fully protect from biliary reflux.

We have demonstrated that all four bile acids commonly identified in human refluxate have a role to some degree in airway EMT pathways. At each stage in the EMT process, we demonstrated characteristic changes with bile acid challenges on primary tracheal epithelial cells. Increased expression of TGF‐β1 is considered the master switch of EMT. E‐cadherin is critical in epithelial polarity maintenance and therefore the development of tight junctions. Acquisition of the mesenchymal phenotype is characterized by an increase in the mesenchymal marker, fibronectin, which plays a role in tissue repair, cell migration and adhesion, and a number of other processes.^19^ EMT is also characterized by expression of MMP‐9, a type IV collagenase and part of the EMT proteome that degrades basement membranes. Collagen type IV forms the reticular basement membrane upon which epithelial cells lie. By disrupting the basement membrane of airway epithelium, MMPs can cause inflammation, translocation, and further EMT,^17^ as marked by procollagen production from challenged epithelial cells.

Our findings show that E‐cadherin gene expression levels did not decrease after stimulation with 1 μmol/L LCA, which may represent a threshold effect. The concentration of BAs that reach the upper airway may be at high concentration, however. It has been shown that the mean levels of bile acids in patients with LPR were 2.1 ± 3.0 µmol/L,^10^ and that levels of total bile acids of > 10 µmol/L were measured in individual patients.^10^

The results of this study indicate that RT‐PCR analysis of E‐cadherin expression revealed a significant increase at both concentrations of DCA, showing a negative concentration‐dependency effect. We think this is of potential significance. One speculation we could make is that the challenge of epithelial cells by some individual bile acids may augment epithelial marker expression.

The results of this study indicate that fibronectin gene expression levels did not increase after stimulation with 10 μmol/L DCA, which contrasted with a significant upregulation with 1 μmol/L DCA. Of note, it has been shown that bile acids inhibit intracellular function as they accumulate. This does not kill the cell but reduces the rate of all cellular processes such as protein synthesis.^20^ Overall, our data makes a case for further studies of an underresearched area and for the investigation of individual bile acids because our work shows clear differences in the response of PTECs to the different bile acids that we evaluated.

This is also illustrated by our finding of procollagen protein after stimulation of human subglottic cells with DCA at 1 μmol/L and CA at 1 and 10 µ μmol/L, contrasting with our data for CDCA 1 and 10 µmol/L, in which procollagen did not increase significantly.

Our findings are further supported by Karagiannidis et al.,^21^ who confirmed increased TGF‐β1, a key EMT marker, in benign tracheal stenosis biopsies. Further studies have demonstrated links between aspiration of gastric refluxate and progression of a number of lower airway fibrotic diseases, including cystic fibrosis, lung transplant rejection, chronic obstructive pulmonary disease, and pulmonary fibrosis.^12^, ^18^ Furthermore, EMT is strongly implicated in laryngotracheal malignancies, and further investigation of the role of bile acids in these cancers is needed.^22^

Cells were also stimulated with 5 ng/mL TGF‐β1 as a positive control in our study. This followed preliminary work in which we established that 5 ng/mL was an optimum concentration of TGF‐β1 to stimulate primary PTECs and cell lines. Published studies also suggest that 5 ng/mL of TGF‐β1 induces morphological changes in immortalized cell lines.^23^, ^24^, ^25^ The mechanism by which TGF‐β1 upregulates MMP‐9 and procollagen has not been fully established. The signal‐inducing flow initiated by the binding of TGF‐β1 to its receptors may be directly responsible.^26^, ^27^ Moreover, TGF‐β1 may upregulate some mediators that regulate MMP‐9 (e.g., nitric oxide) that can increase MMP‐9 activity.^28^

This is an in vitro study, and has obvious limitations for direct extrapolation into the in vivo environment. There is currently no clear evidence quantifying the concentrations of individual bile acids in the laryngopharynx or trachea during or after reflux events. We used concentrations that were maximally stimulating without any significant loss of cell viability, and that have previously been demonstrated to be physiologically relevant in LPR patients in whom total bile acid levels of up to 10 umol/L were measured.

Further investigations of the dose response relationship over a broader range of concentrations would thus be of interest in future studies, combined with measurements of individual bile acids in the upper airway.

## CONCLUSION

We have demonstrated that bile acids can induce markers of EMT in PTECs, a process that may underlie the development of LTS because bile acids come in contact with this region of the airway in reflux disease. This in vitro study suggests a potential causative or synergistic role of bile acids in the development of acquired LTS. Crucially, biliary reflux is a highly treatable target in those at high risk of development of LTS, indicating a potential translational significance of our findings.
